# Crystal structure of a silver-, cobalt- and iron-based phosphate with an alluaudite-like structure: Ag_1.655_Co_1.64_Fe_1.36_(PO_4_)_3_

**DOI:** 10.1107/S205698901700740X

**Published:** 2017-05-26

**Authors:** Adam Bouraima, Thomas Makani, Abderrazzak Assani, Mohamed Saadi, Lahcen El Ammari

**Affiliations:** a Laboratoire de Chimie du Solide Appliquée, Faculty of Sciences, Mohammed V University in Rabat, Avenue Ibn Battouta, BP 1014, Rabat, Morocco; b Département de Chimie, Faculté des Sciences, Université des Sciences et Techniques de Masuku, BP 943, Franceville, Gabon

**Keywords:** crystal structure, Ag_1.655_Co_1.64_Fe_1.36_(PO_4_)_3_, transition metal phosphate, solid-state reaction synthesis, alluaudite-like structure

## Abstract

The new silver-, cobalt- and iron-based phosphate, silver cobalt iron tris(ortho­phosphate), Ag_1.655_Co_1.64_Fe_1.36_(PO_4_)_3_, was synthesized by solid-state reactions. Its structure is isotypic to that of Na_2_Co_2_Fe(PO_4_)_3_, and belongs to the alluaudite family, with a partial cationic disorder, the Ag^I^ atoms being located on an inversion centre and twofold rotation axis sites (Wyckoff positions 4*a* and 4*e*), with partial occupancies of 0.885 (2) and 0.7688 (19), respectively. One of the two P atoms in the asymmetric unit completely fills one 4*e* site while the Co and Fe atoms fill another 4*e* site, with partial occupancies of 0.86 (5) and 0.14 (5), respectively. The remaining Co^2+^ and Fe^3+^ cations are distributed on a general position, 8*f*, in a 0.39 (4):0.61 (4) ratio. All O atoms and the other P atoms are in general positions. The structure is built up from zigzag chains of edge-sharing [*M*O_6_] (*M* = Fe/Co) octa­hedra stacked parallel to [101]. These chains are linked together through PO_4_ tetra­hedra, forming polyhedral sheets perpendicular to [010]. The resulting framework displays two types of channels running along [001], in which the Ag^I^ atoms (coordination number eight) are located.

## Abstract 2

The transition metal orthophosphate Ag_1.655_Co_1.64_Fe_1.36_(PO_4_)_3_ crystallizes in an alluaudite-type structure. The chains characterizing the alluaudite structure are built up from edge-sharing [CoO_6_] and [FeO_6_] octa­hedra linked together by PO_4_ tetra­hedra. The Ag^+^ cations are located in two types of channels in the resulting framework.

## Chemical context   

Compounds belonging to the large alluaudite structural family (Moore, 1971 ▸; Moore & Ito, 1979 ▸; Hatert *et al.*, 2000 ▸, 2004 ▸) have been of continuing inter­est owing to their open-framework architecture, with hexa­gonal-shaped channels, and their physical properties. This fact is amply justified by their practical applications, for example as corrosion inhibitors, passivators of metal surfaces, and catalysts (Korzenski *et al.*, 1999 ▸). In addition, inter­est in alluaudite phosphates with monovalent cations has continued to grow in the electrochemical field, where they have applications as positive electrodes in lithium and sodium batteries (Trad *et al.*, 2010 ▸). Accordingly, our attention is mostly focused on the elaboration and structural characterization of new alluaudite-type phosphates within the *A*_2_O–*M*O–P_2_O_5_ systems (*A* = monovalent cation *M* = divalent cation). For instance, most recently, the hydro­thermal investigation of the Na_2_O–*M*O–P_2_O_5_ pseudo-ternary system has allowed the isolation of the sodium- and magnesium-based alluaudite phosphate NaMg_3_(PO_4_)(HPO_4_)_2_ (Ould Saleck *et al.*, 2015 ▸). On the other hand, within the Na_2_O–CoO–Fe_2_O_3_–P_2_O_5_ and Na_2_O–ZnO–Fe_2_O_3_–P_2_O_5_ pseudo-quaternary systems, solid-state synthesis has allowed Na_2_Co_2_Fe(PO_4_)_3_ (Bouraima *et al.*, 2015 ▸) and Na_1.67_Zn_1.67_Fe_1.33_(PO_4_)_3_ (Khmiyas *et al.*, 2015 ▸) to be obtained. With the same objective, a new silver-, cobalt- and iron-based alluaudite-type phosphate, namely Ag_1.655_Co_1.64_Fe_1.36_(PO_4_)_3_, has been synthesized by means of solid-state reactions and characterized by single crystal X-ray diffraction.

## Structural commentary   

**Figure 1:**
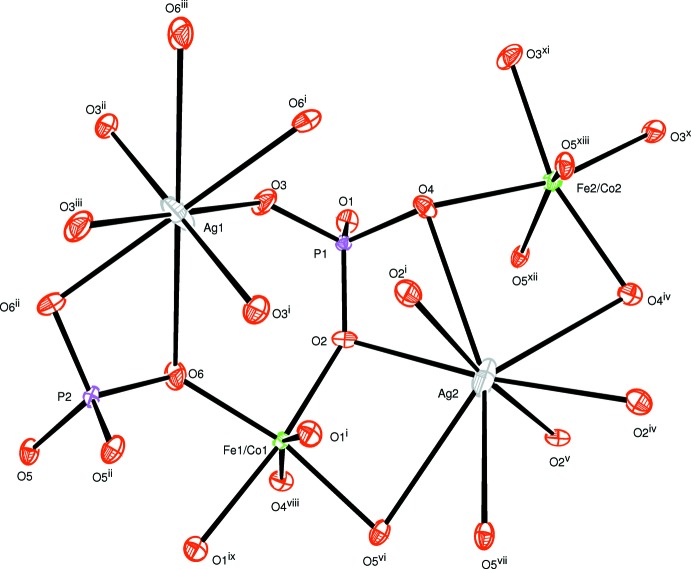
The principal building units in the structure of the title compound. Displacement ellipsoids are drawn at the 50% probability level. [Symmetry codes: (i) *x*, −*y* + 1, *z* + 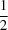; (ii) −*x* + 1, *y*, −*z* + 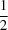; (iii) −*x* + 1, −*y* + 1, −*z* + 1; (iv) −*x* + 2, *y*, −*z* + 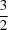; (v) −*x* + 2, −*y* + 1, −*z* + 1; (vi) *x* + 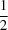, −*y* + 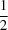, *z* + 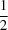; (vii) −*x* + 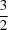, −*y* + 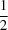, −*z* + 1; (viii) *x*, −*y* + 1, *z* − 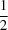; (ix) −*x* + 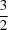, *y* − 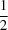, −*z* + 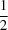; (*x*) *x* + 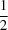, −*y* + 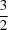, *z* + 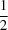; (xi) −*x* + 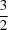, −*y* + 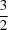, −*z* + 1; (xii) −*x* + 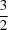, *y* + 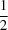, −*z* + 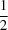; (xiii) *x* + 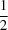, *y* + 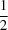, *z* + 1.]

**Figure 2:**
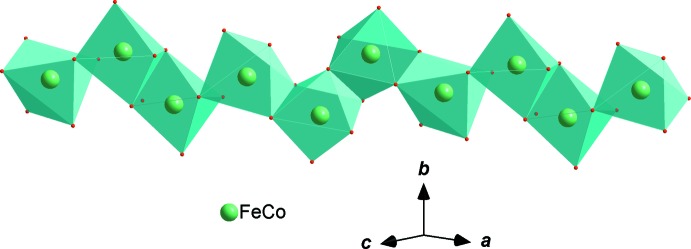
Edge-sharing [(Fe/Co)O_6_] octa­hedra forming a layer parallel to [101].

**Figure 3:**
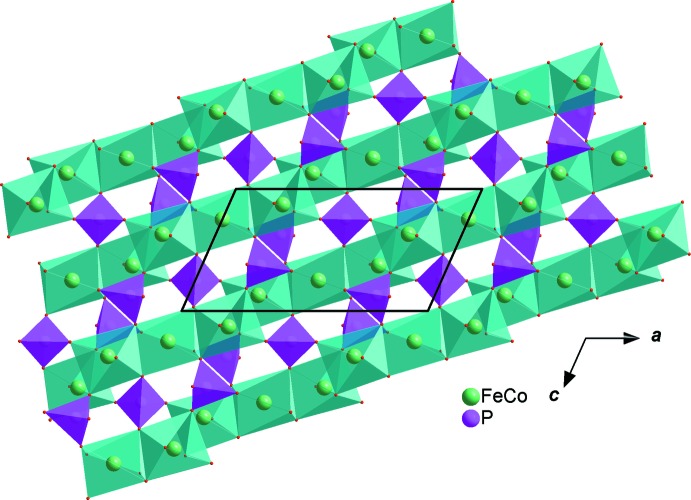
A view along [010], showing a layer resulting from the connection of chains *via* vertices of PO_4_ tetra­hedra and [FeO_6_] octa­hedra.

**Figure 4:**
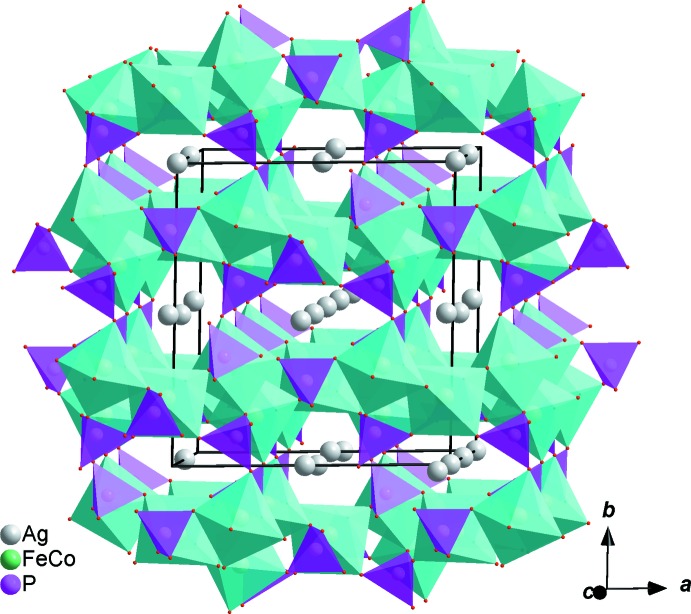
Polyhedral representation of Ag_1.655_Co_1.64_Fe_1.36_(PO_4_)_3_, showing channels running along [001].

In the new isolated compound, either cobalt or iron atoms are distributed in the two octa­hedral sites while the phosphorus atoms are tetra­hedrally coordinated, as shown in Fig. 1 ▸. The structure is built up from two edge-sharing [(Co1/Fe1)O_6_] octa­hedra, leading to the formation of [(Co1/Fe1)_2_O_10_] dimers. Those dimers are connected by a common edge to [(Fe2/Co2)O_6_] octa­hedra, forming an infinite chain (Fig. 2 ▸). The junction between these chains is ensured by sharing vertices with the PO_4_ tetra­hedra so as to form an open layer perpendicular to [010] (Fig. 3 ▸). The three-dimensional framework resulting from the stacking of the sheets along the *b*-axis direction delimits channels parallel to [001] in which the Ag^+^ cations are accommodated, as shown in Fig. 4 ▸.

## Comparison with a related structure   

It is worth mentioning that the distribution of metallic cations observed in the case of the silver–cobalt–iron-based phosphate is not encountered in the sodium homologue. Hence, in the title silver-based phosphate, the octa­hedral *M*1 site (Wyckoff position 8*f*) is occupied to 60% by Fe1 and to 40% by Co1. The octa­hedrally surrounded *M*2 site (Wyckoff position 4*e*) is essentially occupied by Fe2 atoms (43%) along with a small amount of Co2 (7%). However, in the Na_2_Co_2_Fe(PO_4_)_3_ phosphate, the *M*1 and *M*2 sites are entirely occupied by Fe and Co atoms, respectively. For the mixed sites, the occupancy rate was refined without any constraint. The results of the refinements are in good agreement with the electrical neutrality of the compound and calculations of the bond-valence sums of the atoms in the structure (Brown & Altermatt, 1985 ▸). Accordingly, in the silver-based phosphate, the cations at the *M*1 site form double octahedra [(Fe1/Co1)_2_O_10_] alternating with [(Fe2/Co2)O_6_] octa­hedra, while in the sodium homologue phosphate, the obtained [Co_2_O_10_] double octahedra alternate with [FeO_6_] octa­hedra (Fig. 4 ▸). Moreover, both the Ag1 and Ag2 atoms are located in channels, surrounded by eight oxygen atoms with Ag1—O bond lengths between 2.3320 (13) Å and 2.9176 (13) Å, whereas Ag2—O bond lengths are in the range 2.4733 (13)–2.9035 (12) Å. The structure of the title phosphate is isotypic to that of Na_2_Co_2_Fe(PO_4_)_3_ (Bouraima *et al.*, 2015 ▸) and Na_1.67_Zn_1.67_Fe_1.33_(PO_4_)_3_ (Khmiyas *et al.*, 2015 ▸).

## Synthesis and crystallization   

The title compound was isolated from solid-state reactions in air by mixing nitrates of silver, cobalt and iron with phospho­ric acid. The various precursors are taken in the molar ratio Ag:Co:Fe:P = 2:2:1:3. The mixture was stirred at room temperature overnight. After different heat treatments in a platinum crucible at up to 873 K, the reaction mixture was heated to the melting temperature of 1221 K. The molten product was then cooled to room temperature at a rate of 5 K h^−1^. Brown homogeneous crystals corresponding to the title compound of a suitable size for X-ray diffraction were obtained.

## Refinement   

Crystal data, data collection and structure refinement details are summarized in Table 1 ▸. The maximum and minimum residual electron densities in the final Fourier map are 0.68 and 0.55 Å from Ag1 and Ag2, respectively.

## Supplementary Material

Crystal structure: contains datablock(s) I. DOI: 10.1107/S205698901700740X/hp2074sup1.cif

Structure factors: contains datablock(s) I. DOI: 10.1107/S205698901700740X/hp2074Isup2.hkl

CCDC reference: 1551181

Additional supporting information:  crystallographic information; 3D view; checkCIF report

## Figures and Tables

**Table 1:** Experimental details

Crystal data	
Chemical formula	Ag_1.655_Co_1.64_Fe_1.36_(PO_4_)_3_
*M*_r_	2544.10
Crystal system, space group	Monoclinic, *C*2/*c*
Temperature (K)	296
*a*, *b*, *c* (Å)	11.8680 (3), 12.5514 (3), 6.4386 (2)
β (°)	114.012 (1)
*V* (Å^3^)	876.09 (4)
*Z*	1
Radiation type	Mo *K*α
μ (mm^−1^)	9.51
Crystal size (mm)	0.31 × 0.26 × 0.22

Data collection	
Diffractometer	Bruker X8 APEX
Absorption correction	Multi-scan (*SADABS*; Krause *et al.*, 2015 ▸)
*T*_min_, *T*_max_	0.066, 0.124
No. of measured, independent and observed [*I* > 2σ(*I*)] reflections	13097, 2137, 2079
*R*_int_	0.030
(sin θ/λ)_max_ (Å^−1^)	0.833

Refinement	
*R*[*F* ^2^ > 2σ(*F* ^2^)], *wR*(*F* ^2^), *S*	0.020, 0.047, 1.19
No. of reflections	2137
No. of parameters	99
Δρ_max_, Δρ_min_ (e Å^−3^)	1.47, −0.92

Computer programs: *APEX2* aand *SAINT* (Bruker, 2009 ▸), *SHELXT2014* (Sheldrick, 2015*a* ▸), *SHELXL2014* (Sheldrick, 2015*b* ▸), *ORTEP-3 for Windows* (Farrugia, 2012 ▸), *DIAMOND* (Brandenburg, 2006 ▸) and *publCIF* (Westrip, 2010 ▸).

## supplementary crystallographic information

### Crystal data

**Table:**

Ag_1.655_Co_1.64_Fe_1.36_(PO_4_)_3_	*F*(000) = 1194
*M**_r_* = 2544.10	*D*_x_ = 4.822 Mg m^−^^3^
Monoclinic, *C*2/*c*	Mo *K*α radiation, λ = 0.71073 Å
*a* = 11.8680 (3) Å	Cell parameters from 2137 reflections
*b* = 12.5514 (3) Å	θ = 3.3–36.3°
*c* = 6.4386 (2) Å	µ = 9.51 mm^−^^1^
β = 114.012 (1)°	*T* = 296 K
*V* = 876.09 (4) Å^3^	Block, brown
*Z* = 1	0.31 × 0.26 × 0.22 mm

### Data collection

**Table:**

Bruker X8 APEX diffractometer	2137 independent reflections
Radiation source: fine-focus sealed tube	2079 reflections with *I* > 2σ(*I*)
Graphite monochromator	*R*_int_ = 0.030
φ and ω scans	θ_max_ = 36.3°, θ_min_ = 3.3°
Absorption correction: multi-scan (SADABS; Krause *et al.*, 2015)	*h* = −19→19
*T*_min_ = 0.066, *T*_max_ = 0.124	*k* = −20→20
13097 measured reflections	*l* = −10→10

### Refinement

**Table:**

Refinement on *F*^2^	0 restraints
Least-squares matrix: full	*w* = 1/[σ^2^(*F*_o_^2^) + (0.0121*P*)^2^ + 3.2851*P*] where *P* = (*F*_o_^2^ + 2*F*_c_^2^)/3
*R*[*F*^2^ > 2σ(*F*^2^)] = 0.020	(Δ/σ)_max_ = 0.001
*wR*(*F*^2^) = 0.047	Δρ_max_ = 1.47 e Å^−^^3^
*S* = 1.19	Δρ_min_ = −0.92 e Å^−^^3^
2137 reflections	Extinction correction: SHELXL2014 (Sheldrick, 2015*b*), Fc^*^=kFc[1+0.001xFc^2^λ^3^/sin(2θ)]^-1/4^
99 parameters	Extinction coefficient: 0.00102 (10)

### Special details

**Table:**

Geometry. All esds (except the esd in the dihedral angle between two l.s. planes) are estimated using the full covariance matrix. The cell esds are taken into account individually in the estimation of esds in distances, angles and torsion angles; correlations between esds in cell parameters are only used when they are defined by crystal symmetry. An approximate (isotropic) treatment of cell esds is used for estimating esds involving l.s. planes.

### Fractional atomic coordinates and isotropic or equivalent isotropic displacement parameters (Å^2^)

**Table:**

	*x*	*y*	*z*	*U*_iso_*/*U*_eq_	Occ. (<1)
Ag1	0.5000	0.5000	0.5000	0.01952 (7)	0.885 (2)
Ag2	1.0000	0.48916 (3)	0.7500	0.02408 (10)	0.7688 (19)
Fe1	0.78227 (2)	0.34311 (2)	0.37115 (3)	0.00565 (6)	0.61 (4)
Co1	0.78227 (2)	0.34311 (2)	0.37115 (3)	0.00565 (6)	0.39 (4)
Fe2	1.0000	0.76503 (2)	0.7500	0.00714 (8)	0.14 (5)
Co2	1.0000	0.76503 (2)	0.7500	0.00714 (8)	0.86 (5)
P1	0.76272 (3)	0.61138 (3)	0.37428 (6)	0.00502 (8)	
P2	0.5000	0.28909 (4)	0.2500	0.00535 (10)	
O1	0.77807 (11)	0.67841 (10)	0.18620 (19)	0.00908 (19)	
O2	0.81856 (12)	0.49999 (9)	0.3820 (2)	0.0112 (2)	
O3	0.62598 (11)	0.60711 (11)	0.3280 (2)	0.0135 (2)	
O4	0.83676 (12)	0.66524 (9)	0.60788 (19)	0.00917 (19)	
O5	0.45841 (10)	0.21873 (10)	0.03381 (18)	0.00821 (19)	
O6	0.60284 (12)	0.36401 (10)	0.2530 (2)	0.0122 (2)	

### Atomic displacement parameters (Å^2^)

**Table:**

	*U*^11^	*U*^22^	*U*^33^	*U*^12^	*U*^13^	*U*^23^
Ag1	0.02917 (14)	0.00879 (9)	0.01123 (10)	−0.00389 (7)	−0.00138 (8)	−0.00128 (6)
Ag2	0.01069 (12)	0.02795 (16)	0.02519 (15)	0.000	−0.00133 (10)	0.000
Fe1	0.00485 (9)	0.00657 (9)	0.00575 (9)	0.00037 (6)	0.00240 (6)	0.00058 (6)
Co1	0.00485 (9)	0.00657 (9)	0.00575 (9)	0.00037 (6)	0.00240 (6)	0.00058 (6)
Fe2	0.00620 (12)	0.00833 (13)	0.00796 (13)	0.000	0.00398 (10)	0.000
Co2	0.00620 (12)	0.00833 (13)	0.00796 (13)	0.000	0.00398 (10)	0.000
P1	0.00488 (15)	0.00490 (15)	0.00521 (15)	0.00006 (11)	0.00198 (11)	0.00022 (10)
P2	0.00397 (19)	0.0071 (2)	0.00466 (19)	0.000	0.00138 (16)	0.000
O1	0.0101 (5)	0.0112 (5)	0.0062 (4)	−0.0001 (4)	0.0036 (4)	0.0020 (3)
O2	0.0118 (5)	0.0070 (4)	0.0144 (5)	0.0024 (4)	0.0048 (4)	−0.0013 (4)
O3	0.0067 (4)	0.0125 (5)	0.0221 (6)	0.0007 (4)	0.0066 (4)	0.0031 (4)
O4	0.0127 (5)	0.0083 (4)	0.0062 (4)	−0.0011 (4)	0.0034 (4)	−0.0013 (3)
O5	0.0064 (4)	0.0124 (5)	0.0053 (4)	−0.0011 (4)	0.0018 (3)	−0.0017 (3)
O6	0.0085 (5)	0.0119 (5)	0.0166 (5)	−0.0036 (4)	0.0054 (4)	0.0007 (4)

### Geometric parameters (Å, º)

**Table:**

Ag1—O6^i^	2.3320 (13)	Fe1—O4^viii^	2.0481 (12)
Ag1—O6^ii^	2.3320 (13)	Fe1—O1^i^	2.0669 (11)
Ag1—O3^i^	2.4356 (14)	Fe1—O5^vi^	2.0705 (12)
Ag1—O3^ii^	2.4356 (14)	Fe1—O1^ix^	2.1695 (12)
Ag1—O3^iii^	2.5724 (13)	Fe2—O3^x^	2.1099 (13)
Ag1—O3	2.5725 (13)	Fe2—O3^xi^	2.1099 (13)
Ag1—O6^iii^	2.9176 (13)	Fe2—O5^xii^	2.1575 (11)
Ag1—O6	2.9176 (13)	Fe2—O5^xiii^	2.1575 (11)
Ag2—O2^iv^	2.4733 (13)	Fe2—O4^iv^	2.1717 (12)
Ag2—O2	2.4733 (13)	Fe2—O4	2.1717 (12)
Ag2—O2^i^	2.6204 (13)	P1—O3	1.5270 (13)
Ag2—O2^v^	2.6204 (13)	P1—O2	1.5393 (12)
Ag2—O4	2.8341 (12)	P1—O1	1.5451 (12)
Ag2—O4^iv^	2.8341 (12)	P1—O4	1.5543 (12)
Ag2—O5^vi^	2.9035 (13)	P2—O6^ii^	1.5346 (13)
Ag2—O5^vii^	2.9035 (12)	P2—O6	1.5346 (13)
Fe1—O6	1.9656 (13)	P2—O5^ii^	1.5498 (12)
Fe1—O2	2.0108 (12)	P2—O5	1.5498 (12)
			
O6^i^—Ag1—O6^ii^	180.00 (4)	O2—Ag2—O1^i^	64.62 (4)
O6^i^—Ag1—O3^i^	80.58 (4)	O2^i^—Ag2—O1^i^	49.21 (3)
O6^ii^—Ag1—O3^i^	99.42 (4)	O2^v^—Ag2—O1^i^	136.08 (3)
O6^i^—Ag1—O3^ii^	99.42 (4)	O4—Ag2—O1^i^	92.94 (3)
O6^ii^—Ag1—O3^ii^	80.58 (4)	O4^iv^—Ag2—O1^i^	162.54 (3)
O3^i^—Ag1—O3^ii^	180.0	O5^vi^—Ag2—O1^i^	52.36 (3)
O6^i^—Ag1—O3^iii^	108.21 (5)	O5^vii^—Ag2—O1^i^	56.78 (3)
O6^ii^—Ag1—O3^iii^	71.79 (5)	O2^iv^—Ag2—O1^v^	64.62 (4)
O3^i^—Ag1—O3^iii^	66.27 (5)	O2—Ag2—O1^v^	119.96 (4)
O3^ii^—Ag1—O3^iii^	113.73 (5)	O2^i^—Ag2—O1^v^	136.08 (3)
O6^i^—Ag1—O3	71.79 (5)	O2^v^—Ag2—O1^v^	49.21 (3)
O6^ii^—Ag1—O3	108.21 (5)	O4—Ag2—O1^v^	162.54 (3)
O3^i^—Ag1—O3	113.73 (5)	O4^iv^—Ag2—O1^v^	92.94 (3)
O3^ii^—Ag1—O3	66.27 (5)	O5^vi^—Ag2—O1^v^	56.78 (3)
O3^iii^—Ag1—O3	180.0	O5^vii^—Ag2—O1^v^	52.36 (3)
O6^i^—Ag1—O6^iii^	53.64 (5)	O6—Fe1—O2	93.77 (5)
O6^ii^—Ag1—O6^iii^	126.36 (5)	O6—Fe1—O4^viii^	110.10 (5)
O3^i^—Ag1—O6^iii^	95.49 (4)	O2—Fe1—O4^viii^	86.76 (5)
O3^ii^—Ag1—O6^iii^	84.51 (4)	O6—Fe1—O1^i^	86.70 (5)
O3^iii^—Ag1—O6^iii^	68.02 (4)	O2—Fe1—O1^i^	100.62 (5)
O3—Ag1—O6^iii^	111.98 (4)	O4^viii^—Fe1—O1^i^	161.33 (5)
O6^i^—Ag1—O6	126.36 (5)	O6—Fe1—O5^vi^	163.25 (5)
O6^ii^—Ag1—O6	53.64 (5)	O2—Fe1—O5^vi^	101.04 (5)
O3^i^—Ag1—O6	84.51 (4)	O4^viii^—Fe1—O5^vi^	78.79 (5)
O3^ii^—Ag1—O6	95.49 (4)	O1^i^—Fe1—O5^vi^	82.95 (5)
O3^iii^—Ag1—O6	111.98 (4)	O6—Fe1—O1^ix^	80.26 (5)
O3—Ag1—O6	68.02 (4)	O2—Fe1—O1^ix^	171.95 (5)
O6^iii^—Ag1—O6	180.0	O4^viii^—Fe1—O1^ix^	90.22 (4)
O2^iv^—Ag2—O2	173.70 (6)	O1^i^—Fe1—O1^ix^	84.52 (5)
O2^iv^—Ag2—O2^i^	101.33 (4)	O5^vi^—Fe1—O1^ix^	85.66 (5)
O2—Ag2—O2^i^	78.34 (4)	O3^x^—Fe2—O3^xi^	80.97 (7)
O2^iv^—Ag2—O2^v^	78.34 (4)	O3^x^—Fe2—O5^xii^	91.27 (5)
O2—Ag2—O2^v^	101.33 (4)	O3^xi^—Fe2—O5^xii^	112.81 (5)
O2^i^—Ag2—O2^v^	174.04 (5)	O3^x^—Fe2—O5^xiii^	112.81 (5)
O2^iv^—Ag2—O4	118.73 (4)	O3^xi^—Fe2—O5^xiii^	91.27 (5)
O2—Ag2—O4	55.50 (4)	O5^xii^—Fe2—O5^xiii^	148.74 (7)
O2^i^—Ag2—O4	61.33 (4)	O3^x^—Fe2—O4^iv^	85.08 (5)
O2^v^—Ag2—O4	113.50 (4)	O3^xi^—Fe2—O4^iv^	164.39 (5)
O2^iv^—Ag2—O4^iv^	55.50 (4)	O5^xii^—Fe2—O4^iv^	74.29 (4)
O2—Ag2—O4^iv^	118.73 (4)	O5^xiii^—Fe2—O4^iv^	87.71 (4)
O2^i^—Ag2—O4^iv^	113.50 (4)	O3^x^—Fe2—O4	164.39 (5)
O2^v^—Ag2—O4^iv^	61.33 (4)	O3^xi^—Fe2—O4	85.08 (5)
O4—Ag2—O4^iv^	77.51 (5)	O5^xii^—Fe2—O4	87.71 (4)
O2^iv^—Ag2—O5^vi^	114.87 (4)	O5^xiii^—Fe2—O4	74.29 (4)
O2—Ag2—O5^vi^	71.23 (4)	O4^iv^—Fe2—O4	109.56 (7)
O2^i^—Ag2—O5^vi^	101.56 (4)	O3—P1—O2	112.57 (7)
O2^v^—Ag2—O5^vi^	83.86 (4)	O3—P1—O1	108.72 (7)
O4—Ag2—O5^vi^	125.84 (3)	O2—P1—O1	109.47 (7)
O4^iv^—Ag2—O5^vi^	144.70 (3)	O3—P1—O4	109.98 (7)
O2^iv^—Ag2—O5^vii^	71.23 (4)	O2—P1—O4	107.31 (7)
O2—Ag2—O5^vii^	114.87 (4)	O1—P1—O4	108.73 (7)
O2^i^—Ag2—O5^vii^	83.86 (4)	O6^ii^—P2—O6	104.43 (10)
O2^v^—Ag2—O5^vii^	101.56 (4)	O6^ii^—P2—O5^ii^	108.75 (7)
O4—Ag2—O5^vii^	144.70 (3)	O6—P2—O5^ii^	112.14 (7)
O4^iv^—Ag2—O5^vii^	125.84 (3)	O6^ii^—P2—O5	112.14 (7)
O5^vi^—Ag2—O5^vii^	52.03 (4)	O6—P2—O5	108.75 (7)
O2^iv^—Ag2—O1^i^	119.96 (4)	O5^ii^—P2—O5	110.52 (9)

Symmetry codes: (i) *x*, −*y*+1, *z*+1/2; (ii) −*x*+1, *y*, −*z*+1/2; (iii) −*x*+1, −*y*+1, −*z*+1; (iv) −*x*+2, *y*, −*z*+3/2; (v) −*x*+2, −*y*+1, −*z*+1; (vi) *x*+1/2, −*y*+1/2, *z*+1/2; (vii) −*x*+3/2, −*y*+1/2, −*z*+1; (viii) *x*, −*y*+1, *z*−1/2; (ix) −*x*+3/2, *y*−1/2, −*z*+1/2; (x) *x*+1/2, −*y*+3/2, *z*+1/2; (xi) −*x*+3/2, −*y*+3/2, −*z*+1; (xii) −*x*+3/2, *y*+1/2, −*z*+1/2; (xiii) *x*+1/2, *y*+1/2, *z*+1.
